# Construction of an Electrochemical Sensor Based on Carbon Nanotubes/Gold Nanoparticles for Trace Determination of Amoxicillin in Bovine Milk

**DOI:** 10.3390/s16010056

**Published:** 2016-01-20

**Authors:** Aliyu Muhammad, Nor Azah Yusof, Reza Hajian, Jaafar Abdullah

**Affiliations:** 1 Department of Chemistry, Faculty of Science, Universiti Putra Malaysia, 43400 Selangor, Malaysia; amnakowa@yahoo.com (A.M.); jafar@ upm.edu.my (J.A.); 2 Institute of Advanced Technology, Universiti Putra Malaysia, 43400 Selangor, Malaysia

**Keywords:** amoxicillin, bovine milk, electrochemical sensor, MWCNTs, ethylediamine, AuNPs

## Abstract

In this work, a novel electrochemical sensor was fabricated for determination of amoxicillin in bovine milk samples by decoration of carboxylated multi-walled carbon nanotubes (MWCNTs) with gold nanoparticles (AuNPs) using ethylenediamine (en) as a cross linker (AuNPs/en-MWCNTs). The constructed nanocomposite was homogenized in dimethylformamide and drop casted on screen printed electrode. Field emission scanning electron microscopy (FESEM), energy dispersive X-Ray (EDX), X-Ray diffraction (XRD) and cyclic voltammetry were used to characterize the synthesized nanocomposites. The results show that the synthesized nanocomposites induced a remarkable synergetic effect for the oxidation of amoxicillin. Effect of some parameters, including pH, buffer, scan rate, accumulation potential, accumulation time and amount of casted nanocomposites, on the sensitivity of fabricated sensor were optimized. Under the optimum conditions, there was two linear calibration ranges from 0.2–10 µM and 10–30 µM with equations of I_pa_ (µA) = 2.88C (µM) + 1.2017; r = 0.9939 and I_pa_ (µA) = 0.88C (µM) + 22.97; r = 0.9973, respectively. The limit of detection (LOD) and limit of quantitation (LOQ) were calculated as 0.015 µM and 0.149 µM, respectively. The fabricated electrochemical sensor was successfully applied for determination of Amoxicillin in bovine milk samples and all results compared with high performance liquid chromatography (HPLC) standard method.

## 1. Introduction

**Figure 1:**
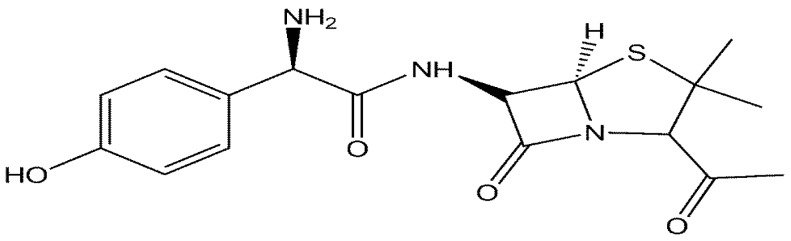
Chemical structure of amoxicillin.

Amoxicillin (2S,5R,6R)-6{[(2R)-2-amino-2-(4-hydroxylphenyl)-acetyl]amino}-3,3-dimethyl-7-oxo-4-thia-1-azabicyclo[3.2.0]heptane-2-carboxylic acid), belongs to the β-lactam group of antibiotics and same as all other members of this group, Amoxicillin structure (Figure 1) contains a β-lactam ring which is responsible for its anti-bacterial property but differs from other members of the group by the side-chain which account for the major differences in their chemical and pharmacological properties [1].

Amoxicillin is used to treat bacterial infections in both human and animals [2], being one of the most commonly used antibiotics in the treatment of bacterial infections in dairy cattle. Amoxicillin residues are found in different dairy products (*i.e*., milk and yogurt) [3]. The residues enter the milk supply whenever withdrawal periods are not strictly adhered to or when a cow retains the residues in its system for an extraordinary length of time. Presence of these residues in milk holds the risk of undesirable health hazards for the consumers, ranging from allergic reactions, development of mechanisms of antibiotic resistance and other related diseases [4]. To avoid these consequences, milk of dairy origin need to be analyzed for the presence of these residues before consumption. 

Various analytical methods have been reported for the detection and separation of Amoxicillin including high performance liquid chromatography (HPLC) [5,6], and capillary electrophoresis [7,8], These methods have the advantage of accuracy but they also have some limitations such as being expensive, large size and time consuming. Some of these methods (e.g., HPLC) required large amount of high purity organic solvents, long equilibration and derivatization treatment. Electrochemical techniques that use in sensor technology are characterized by simplicity, high sensitivity, good stability, low cost instrumentation and portability for on-site monitoring have received tremendous attention. The most common materials use in sensor technology is nanocomposites which are combinations of nanomaterials with other molecules or nanoscaled materials, such as nanoparticles (NPs) or nanotubes. Among these nanocomposites, composites of gold nanoparticles and carbon nanotubes are of great interest.

Carbon nanotubes (CNTs) have captured the interest of researchers worldwide due to their small size with large surface area, high electrical conductivity, chemical stability and mechanical strength [9,10]. CNTs allowed an increase binding site for detection of analytes whenever they are use as surface modifiers for electrodes [11,12]. Gold nanoparticles (AuNPs) attracted greater interest due to their quantized charging/discharging characteristics, electrocatalytic properties, good stability and biocompatibility [13,14]. Nanocomposites of CNTs and AuNPs have the potential to enhance the performance of electrochemical sensors, due to the combination of excellent physical and chemical properties of AuNPs and CNTs [15]. The easy surface modification of gold nanoparticle and the excellent conductivity of carbon nanotube as well as high surface area, point towards a broad range of applications such as biosensing, gas sensing and chemical sensors [16]. Several papers have reported the use of nanocomposites containing AuNPs and CNTs for electrochemical analysis [17]. Hajian and coworkers [13,18] have reported the modification of glassy carbon electrode (GCE) by electrografting ethylenediamine (en) which serves as a linker between separately prepared AuNPs and functionalized multi-walled carbon nanotubes MWCNTs. The fabrication of AuNPs/MWCNTs was based on sandwich modeling where MWCNTs was first drop casted on the surface of GCE and after drying in ambient temperature, ethylenediamine was electrografted by cyclic voltammetry and finally, it was dipped in a solution containing AuNPs for 30 min. 

Here we have reported a novel procedure for bulk scale construction of AuNPs/en-MWCNTs nanocomposite in one step for fabrication of an electrochemical sensor by electroless deposition process using ethylenediamine as a linker for decoration of carbon nanotubes with gold nanoparticles. Construction of the nanocomposite (AuNPs/en-MWCNTs) was based on the chemical reaction of ethylenediamine with carboxylic groups under reflux followed by electrostatically bonding of $AuCl_{4}^{-}$ with amine groups. The conversion of $AuCl_{4}^{-}$ to AuNPs was a chemical process involving sodium citrate as a reducing agent. The constructed nanocomposite in bulk scale can easily be drop casted on the surface of screen printed electrode (SPE) and use after drying in ambient temperature. To the best of our knowledge, there is no documented information on the detection of Amoxicillin in milk samples using AuNPs/en-MWCNTs modified SPE.

## 2. Material and Methods

### 2.1. Reagents and Solutions

MWCNTs with diameter of 7–12 nm, length of 0.5–10 µm, ethylenediamine, sodium citrate and Casein protein were obtained from Sigma-Aldrich (St. Louis, MO, USA). Tetrachloroaurate (III) (99.5%) was supplied from EMSURE (Darmstadt, Germany), the stock solution of hydrogen tetrachloroaurate (III) (HAuCl_4_) was prepared by dissolving an amount equivalent to 0.1% in deionized water. Amoxicillin trihydrate and Lactose monohydrate were obtained from Fluka (St. Louis, MO, USA). The stock solutions of Amoxicillin and Lactose were prepared by dissolving an amount equivalent to 0.001 M in deionized water. Ethylenediamine-ethanol solution (en-ethanol) was prepared by dissolving an amount of ethylenediamine that is equivalent to 0.1 M in ethanol. Milk samples were collected from Putra mart (Serdang, Selangor, Malaysia) and Fresh milk (commercial brand, Johor, Malaysia). 0.1 M Phosphate buffer solution (pH 7.0) was used as supporting electrolyte in all experiments. Milli-Q water was used for preparing all solutions. All other chemicals were of analytical grade and used without further purification.

### 2.2. Apparatus

DropSens 8400 electrochemical system (Metrohm Co., Herisau, Switzerland) was employed for all voltammetric measurements. A three- electrode configuration was employed including a bare SPE modified with AuNPs/en-MWCNTs as working electrode. The structure, composition and morphology of the synthesized nanocomposites were studied using Fourier transform infrared spectroscopy (FTIR) (Perkin Elmer), Field emission scanning electron microscopy coupled with energy dispersive X-ray (FESEM-EDX) (JEOL JSM 7600F) and X-ray diffraction (XRD) (Philips). pH meter (Fisher science education, Waltham, MA, USA) was used to adjust pH of solutions before each measurement. Kubota 2100 centrifuge (Tokyo, Japan) was used for centrifugation. The samples for FTIR were scanned between 500 to 4000 cm^−1^ wavenumber at a resolution of 4 cm^−1^. FESEM images were obtained by placing small amount of the samples onto a piece of double sided tape stuck to a metal stub. The sample for XRD was placed on a flat amorphous silica sample holder. XRD patterns from 10 to 60 degree at 2θ were recorded at room temperature using Cuka radiation (λ = 1.5418 Å).

### 2.3. Real Sample Extraction

The method employed by Moors and Massart [18] was adopted with little modifications, the details are described briefly as follows; 30 mL raw milk was spiked with known concentration of Amoxicillin and vortex for about 2 min at room temperature to allow the equilibration of β- lactams with milk matrix before their extraction, which was then centrifuged at 3000 rpm for 10 min, 10 mL of the defatted milk was deproteinized by adding 10 mL of acetonitrile (ACN) drop wise with continuous vortex mixing and centrifuged for 15 min at 4000 rpm. After centrifugation, 10 mL of the supernatant was collected into a beaker and heated at 60 °C to evaporate ACN until the volume approximately reduced to 1 mL, 1 mL of phosphate buffer solution was immediately added to avoid degradation of the analyte, the mixture was centrifuged at 4000 rpm for 20 min and the supernatant was filtered through 0.22 µm filter paper and used for analysis.

### 2.4. Preparation of AuNPs/en-MWCNTs Sensor

#### 2.4.1. Pretreatment of MWCNTS

The MWCNTs were refluxed in a mixture of concentrated H_2_SO_4_ and HNO_3_ (3:1) for 6 h to attach carboxyl group to carbon nanotubes. The suspension was filtered through 0.22 µm filter, washed with doubly distilled water till pH neutral and dried in oven at 50 °C overnight. The resulting solid is termed as carboxylated multi-walled carbon nanotubes.

#### 2.4.2. Synthesis of AuNPs/en-MWCNTs

**Scheme 1:**
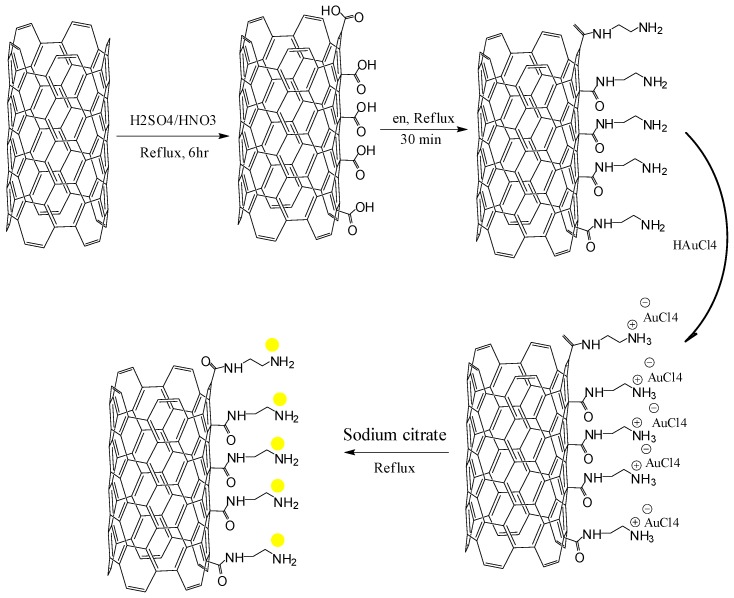
Bulk scale synthetic illustration of AuNPs/en-MWCNTs nanocomposite using ethylenediamine as a cross linker.

10 mg of the carboxylated MWCNTs was added to 25 mL en-ethanol solution (0.1 M) and subjected to reflux for 30 min, 10 mL of HAuCl_4_ (0.01%) was added into the reaction system and further refluxed till boiling. Two milliliters of sodium citrate (1% W/V) was added drop wise into the solution. The mixture was left refluxing for additional 30 min, heating was stopped while stirring continued for 20 min till cooling and the resulting solution was centrifuged at 4000 rpm for 5 min, washed several times with distilled water and dried in an oven at 50 °C overnight. Scheme 1 shows a schematic of the process to fabricate bulk scale nanocomposites of AuNPs/en/MWCNTs under chemical reaction.

#### 2.4.3. Fabrication of AuNPs/en-MWCNTs Based Electrode

The AuNPs/en-MWCNTs suspension was prepared by dispersion of 1 mg AuNPs/en-MWCNTs in 1 mL of dimethylformamide (DMF) using ultrasonic agitation to obtain a relatively stable suspension. Five microliters of the homogeneous suspension was drop casted on the surface of SPE and dried in air to evaporate DMF. MWCNTs/SPE was also prepared following the same procedure. 

The electrochemical properties of the modifiers (*i.e*., MWCNTs and AuNPs) were evaluated by calculating the electro-active surface areas of the electrodes: (a) bare SPE; (b) MWCNTs/SPE; (c) and AuNPs/en/MWCNTs by cyclic voltammetry (CV) in 1.0 mM K_3_Fe(CN)_6_ as a redox probe at different scan rates, the electro-active surface areas were estimated according to Randles–Sevcik equation:

(1) $$\text{Ipa}=(2.69x\;10^{5})\;A.D^{1/2\;}n^{3/2}v^{1/2}C_{o}$$

where I_pa_ is the anodic peak current, n is the number of electron transfers, A = electrode surface area, D = diffusion coefficient (7.6 × 10^−6^ cm^2^·s^−1^), *v* = scan rate and Co is concentration of K_3_Fe(CN)_6_. n = 1. The effective surface area was calculated to be 0.024, 0.034 and 0.046 cm^2^ for bare, MWCNTs and AuNPs/en-MWCNTs modified SPEs, respectively.

### 2.5. Characterizations of AuNPs/en-MWCNTs

The carboxylated and un-carboxylated MWCNTs were characterized by Fourier transmission infrared radiation (FTIR) (Figure S1). Figure S1A shows a broad band at 3421 cm^−1^ and a weak band at 1627 cm^−1^, which are assigned to the asymmetric bending and scissoring vibrations, respectively, resulting from the moisture in the sample [19]. The spectra of the carboxylated MWCNTs (Figure S1B) have an absorption peak at 1796 cm^−1^, which was absent in the un-carboxylated MWCNTs (Figure S1A). This peak at 1796 cm^−1^ corresponds to C=O stretching frequency which arises due to the formation of carboxylic group while the peak at 3427 cm^−1^ correspond to that of O-H stretching vibration [20,21]. The analysis of spectra (Figure S1B) suggest that the peak at 1715 cm^−1^ merges with the 1634 cm^−1^ resulting in its broadening (Figure S1C). The weak peak at 1070 cm^−1^ in Figure S1C is from the C−N stretching vibration of the primary amine end groups [22]. Broadening of the peak at 3440 cm^−1^ further confirms the presence of –NH stretching frequency [23]. These observations confirm the formation of amino functional group on the surface of MWCNTs.

**Figure 2:**
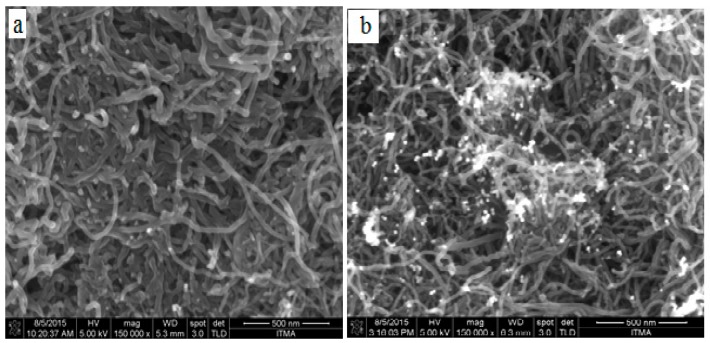
FESEM Images of (**a**) MWCNTs film; and (**b**) AuNPs/en-MWCNTs films.

**Figure 3:**
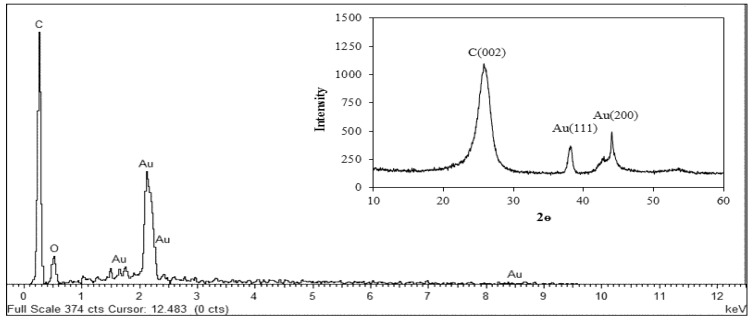
EDX Spectra of AuNPs/en-MWCNTs nanocomposites. Inset: XRD Spectra of the synthesized nanocomposites.

Field emission scanning electron Microscopy (FESEM) was employed to study the surface morphology of the multi-walled carbon nanotubes. The result (Figure 2a) displayed some network-like structures while in Figure 2b many light features were displayed due to the growth of AuNPs which suggest the decoration of gold nanoparticles on the surface of multi-walled carbon nanotubes. The elemental composition of the synthesized nanocomposites was studied using energy dispersive X-ray (EDX). The result (Figure 3) shows the EDX spectrum of the nanocomposites, the element C in the spectrum is basically from the MWCNTs; the element O is oxygen, which is believed to be from COO^−^ group. The presence of Au signals in the spectrum suggested the nanocomposites contained gold nanoparticles. Consequently, the decoration of AuNPs on the MWCNTs, which was shown by FESEM, was confirmed by EDX technique. The composition of the nanocomposites was further studied by X-ray Diffractive (XRD) analysis. The result (Inset Figure 3) presents the XRD patterns of the synthesized nanocomposites, as seen the peaks at 38.20° and 44.40° are assigned to the face centered cubic (fcc) bulk gold planes of (111) and (200), while the diffraction peak at 25.70° is attributed to the graphite crystalline phase of MWCNTs [24,25]. The XRD result indicates that the nanocomposites contained MWCNTs and AuNPs.

### 2.6. Analytical Procedure

The voltammetric response of Amoxicillin was investigated by measuring the oxidation peak current in the potential range of −0.5 to 0.5 V using 0.1 M phosphate buffer (pH 7.0) as the supporting electrolyte. Adsorptive stripping voltammetry was performed by applying accumulation potential of −0.4 V for 180 s and 10 s equilibrium time. Each voltammogram was recorded at scan rate of 0.1 V·s^−1^. All measurements were conducted at room temperature (25 °C ± 1 °C). 

## 3. Results and Discussion

### 3.1. Electrochemical Behavior of Amoxicillin 

**Figure 4:**
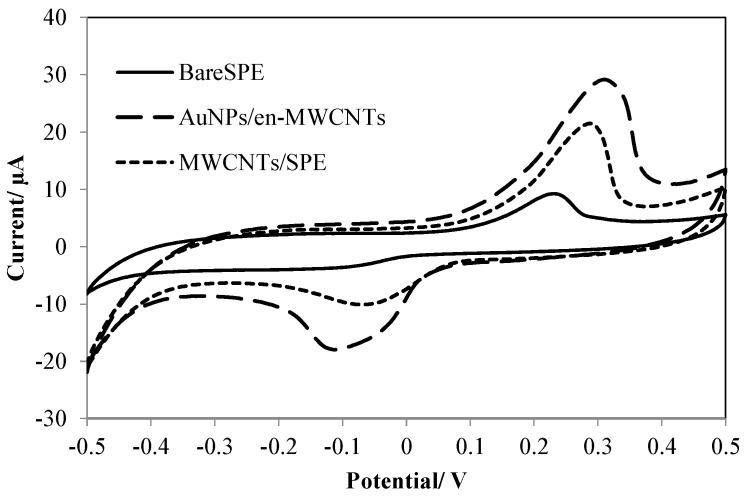
Cyclic voltammograms of 10 µM Amoxicillin on bare SPE and after modification with MWCNTs and AuNPs/en-MWCNTs in phosphate buffer (pH 4.0).

Cyclic voltammetry was used to investigate the electrochemical behavior of Amoxicillin at the modified electrodes. The presence of weak irreversible redox peaks on the surface of the bare SPE (Figure 4), indicating a sluggish electron transfer property of the electrode and lower accumulation of Amoxicillin due to low surface area was observed, while, the peaks at MWCNTs/SPE were higher because of the excellent electro transfer ability and higher surface area of MWCNTs. The sensitivity of the redox peaks enhanced dramatically after decoration of MWCNTs with AuNPs. This shows that the combination of AuNPs and MWCNTs enhanced the electron-transfer rate and surface area due to their noted properties. 

### 3.2. Optimization of Effective Parameters on the Sensitivity of the Electrochemical Sensor

#### 3.2.1. Effect of Supporting Electrolyte

The Effect of pH on the voltammetric response of 10 µM Amoxicillin was studied in the presence of 0.1 M phosphate buffer solution within the pH range of 3–10. By increasing of pH from 3.0, the oxidation peak height increases until it reaches to pH 7.0 then the peak current drastically decreased which clearly indicates that pH 7.0 is the best medium for the voltammetric oxidation of Amoxicillin. A plot of peak potential *versus* pH (Figure S2) had a slope of 0.0592 V, which is close to the theoretical value of 0.06 V, suggesting that the number of protons and electrons involved in the electrochemical oxidation of Amoxicillin are equal. The influence of buffer on the oxidation peak current of Amoxicillin was studied at pH 7.0. Some buffers including citrate, Britton Robinson, and phosphate of equal ionic strength (0.1 M, pH 7.0) were used. The best result with respect to sensitivity was obtained with Phosphate buffer, indicating that the voltammetric oxidation of Amoxicillin is more favorable in phosphate buffer solution. Therefore, phosphate buffer (pH) was chosen as the best supporting electrolyte.

#### 3.2.2. Accumulation Parameters

**Figure 5:**
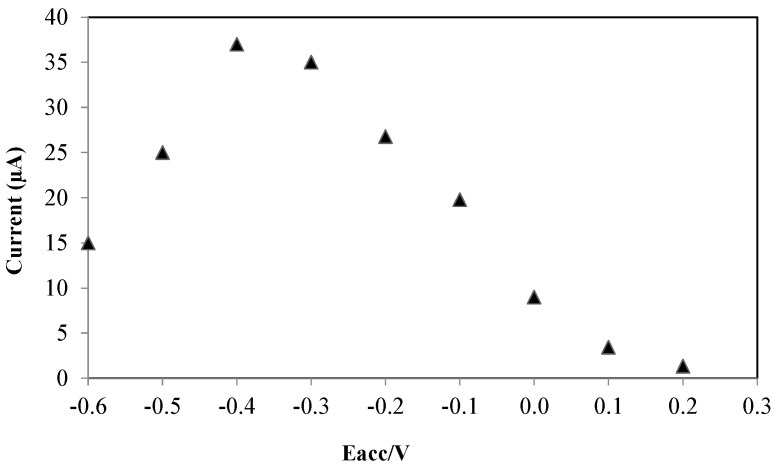
Effect of accumulation potential on the oxidation peak current of Amoxicillin on the surface of AuNPs/en-MWCNTs/SPE. Amoxicillin, 10 µM; Phosphate buffer, pH 7.0; Accumulation time, 60 s.

It is important to optimize the accumulation potential (E_acc_) and accumulation time (t_acc_) when adsorption studies are intended because both parameters can affect the sensitivity of the electrode during electrochemical analysis [26,27]. The effect of accumulation potential on the voltammetric oxidation of Amoxicillin was studied, the result (Figure 5), shows that by variation of E_acc_ from 0.2 to −0.4 V, the peak current increased steadily because the reduced form of Amoxicillin molecules have more adsorptivity on the surface of the modified electrode. But at potentials negative than −0.4 V the peak current decreased because at potentials (<−0.4 V), the layer of nanocomposite on the surface of SPE is unstable during electrolysis of solvent and hydrogen bubbling. Hence, accumulation potential of −0.4 V was considered the optimum value for the voltammetric oxidation of Amoxicillin at the modified electrode. 

**Figure 6:**
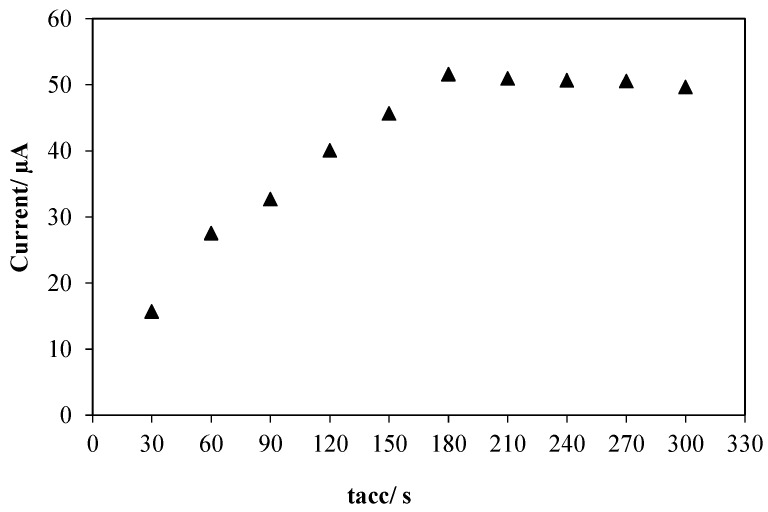
Effect of accumulation time on the oxidation peak current of Amoxicillin on the surface of AuNPs/en-MWCNTs/SPE. Amoxicillin, 10 µM; Phosphate buffer, pH 7.0; Accumulation potential, −0.4 V.

The influence of accumulation time was also investigated in the range of 30–300 s. The result (Figure 6) shows that the peak current increased with increase in accumulation time from 30 to 180 s which can be attributed to the rapid adsorption of Amoxicillin on the surface of electrode. However, the oxidation peak current was leveled off with further increase in accumulation time beyond 180 s. This can be ascribed to the saturation of Amoxicillin on the surface of electrode. Consequently, accumulation time of 180 s was chosen as the optimum value for analysis. 

#### 3.2.3. The Effect of Drop Casted Volume

The drop casting volume of nanocomposites suspension at 1 mg/mL on the voltammetric response of Amoxicillin was investigated (Figure S3). As has been shown, by increasing the volume of nanocomposites from 2.0 to 5.0 µL, the peak current was enhanced dramatically but at volumes more than 5 µL, the sensitivity decreased. This can be attributed to the thickness of the nanocomposites film which blocked the electrical conducting surface of electrode. Therefore, 5 µL was chosen as the optimum amount of nanocomposites which was used for fabrication of the sensor.

#### 3.2.4. Influence of HAuCl_4_ Concentration

The effect of HAuCl_4_ concentration on the peak current of Amoxicillin was carefully studied in the range of 0.001 to 0.05% (W/V) The oxidation peak current of Amoxicillin increased with increasing concentration of HAuCl_4_ from 0.001 to 0.01% (W/V) and then decreased at higher concentrations (Figure S4). This behavior can be attributed to the aggregation of AuNPs, which leads to the decrease in the electroactive surface area. Therefore, 0.01% (W/V) was chosen as the optimum concentration of HAuCl_4_ in the fabrication of AuNPs/en/MWCNTs.

#### 3.2.5. Effect of Scan Rate

The electrochemical behavior of Amoxicillin was studied under different scan rate (10 to 100 mV·s^−1^), The result obtained shows that, as the scan rate increase the oxidation peak current for Amoxicillin increases with a good linear relationship existing between the peak current and scan rate under the equation of (I_pa_ = 563.67*v* + 3.3382; r^2^ = 0.9903). The linear correlation r^2^ = 0.9903 indicates that electrochemical oxidation of Amoxicillin at the modified electrode is under adsorption control.

Furthermore, by plotting log (I_pa_) *vs.* log *v*, a straight line was obtained that can be expressed as: 

(2) $$\text{log}\;(I_{\text{pa}})\left(µA\right)=0.98\log v\left(mV/s\right)-0.2276;\;r^{2}=0.98$$

The slope of 0.98 is close to the theoretical value of 1 showing adsorption control process. 

The number of electrons involve in the electrochemical oxidation of Amoxicillin at modified electrode was determined by Laviron’s equation [28].

(3) $$I_{\text{pa}}=n^{2}F^{2}AГv/4RT$$

where

(4) Q=nFAГ

From Equations (3) and (4), we found that:

(5) $$I_{\text{pa}}=nFQv/4RT$$

Based on the slope of i_pa_ *vs.* *v*, using Equation (5) where n is the number of electrons transferred, F (C·mol^−1^) is the Faraday’s constant, Q (C) is the quantity of charge and *v* (V·s^−1^) is the scan rate, the value of n was estimated to be 0.95, which suggested that one electron is involved in the oxidation reaction. Thus, it can be concluded that the voltammetric oxidation of Amoxicillin at the AuNPs/en-MWCNTs/SPE is accompanied by loss of one proton and one electron which is comparable with previous report [29].

It was observed that the peak potential (Ep) shifted to more positive values with increasing scan rate (*v*). A plot of E_p_ *versus* log *v* yields a straight line with a slope equal to 2.3 RT/(1-α)nF for the anodic peak [28]. By using the slope of the plot the value of α which is the electron transfer coefficient of the voltammetric oxidation of Amoxicillin at the electrode was found to be 0.628. The value of K_0_, which is the electron transfer rate constant, was found to be 946 s^−1^, suggesting that there is high electron promotion process between the electrode surface and the modifier towards oxidation of Amoxicillin.

### 3.3. Figures of Merit

**Figure 7:**
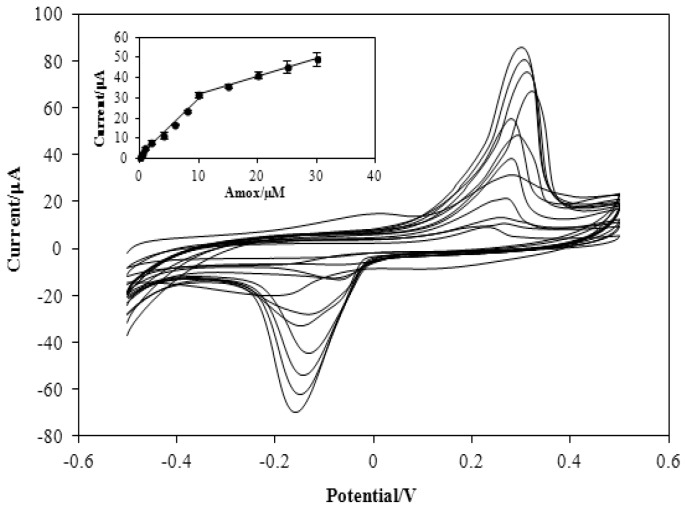
Adsorptive stripping response of different concentration of Amoxicillin at the modified electrode. Inset: Calibration curve of the peak current *versus* concentration of Amoxicillin in the presence of 0.1 M Phosphate buffer (0.1 M, pH 7.0); accumulation time, 180 s; accumulation potential, −0.4 V; and scan rate, 0.1 V·s^−1^.

Adsorptive stripping voltammetric (AdSV) technique was used to investigate the relationship between peak current and concentration of Amoxicillin under the optimized experimental conditions, the results (Figure 7) shows a linear relationship between peak current (I_pa_) and Amoxicillin concentration within the range of 0.2 to 30 µM which is describe by the following linear regression equations: I_pa_ (µA) = 2.88C (µM) + 1.202; r = 0.9939 and I_pa_ (µA) = 0.88C (µM) + 22.97; r= 0.9973 for the range of 0.2–10 µM and 10–30 µM, respectively. The limit of detection (LOD, defined as 3 Sb/m, Sb, standard deviation of blank solution, m, slope of calibration curve) was found to be 0.015 µmol·L^−1^ (n = 3), while, the limit of quantification (LOQ, defined as 10 Sb/m; n =3) was found as 0.149 µmol·L^−1^. The limit of detection and sensitivity for Amoxicillin detection using the proposed method are better compared to other methods that were previously reported (Table 1). The LOD obtained using this fabricated sensor is less than the current legislative MRL of 10 μg/kg (equal to 0.0274 µM) for amoxicillin in milk samples based on Food and Drug Administration (FDA, Silver Spring, MD, USA) [33]. It shows that the constructed sensor has enough sensitivity for detection of amoxicillin residues in dairy products.

**Table 1:** Comparison of the proposed electrochemical sensor with previous reports on the voltammetric determination of amoxicillin.

Electrode	Technique	LDR/µM	LOD/µM	Ref.
Ni/Curcumin/CPE	Amp ^1^, CV	8–100	5	[29]
MWCNT/GCE	AdSV ^2^	0.6–80	0.2	[30]
Glutaraldehyde/GA ^3^/GCE	SWV ^4^	2–25	0.92	[31]
PNI ^5^/CPE	CV	1–200	0.812	[32]
AuNPs/en-MWCNTs/SPE	AdSV	0.2–30	0.015	Present work

^1^ Amperometry; ^2^ Adsorptive stripping voltammetry; ^3^ Glutamic acid; ^4^ Square wave voltammetry; ^5^ Poly(N-vinyl imidazole).

The selectivity of the proposed sensor to the determination of Amoxicillin was evaluated by studying the discrimination against common interferents in bovine milk under optimized experimental conditions. The obtained signal for a fixed concentration of Amoxicillin (10 µmol·L^−1^) was compared with the signal values obtained in the presence of variable concentrations of some β-lactam antibiotics and other possible interferent. The tolerance limit was considered as the maximum concentration of interfering specie that cause an error less than ±5% for the determination of Amoxicillin. The results (Table 2) shows that 50-fold excess concentration of ampicillin, penicillin G, and thiamphenicol, 100-fold concentration of Lactose and Casein protein and 200-fold concentration of K^+^, Na^+^, Ca^2+^, Mg^2+^, Fe^2+^ and Mn^2+^ did not interfere with the voltammetric determination of 10 µmol·L^−1^ Amoxicillin. This study evidenced good selectivity of the fabricated sensor towards Amoxicillin detection. The selectivity of the proposed sensor towards Amoxicillin is based on the fact that Amoxicillin is the only phenolic penicillin and a moderate-spectrum β-lactam antibiotic and the electro-oxidation process occurred at the *p*-hydroxy substituent of Amoxicillin [30,31] which is absent in other members of the β-lactam group and also absent in all other possible interferents. 

**Table 2:** Result of interference studies for the detection of Amoxicillin (10 µM) at optimum conditions.

Specie	Tolerance Limit (Fold)	Signal Change (%)
Ampicillin	50	−4.00
Penicillin G	50	−4.65
Thiamphenicol	50	−4.77
Lactose	100	+0.79
Casein protein	100	−4.72
K^+^, Ca^2+^, Mg^2+^, Zn^2+^, Na^+^, Mn^2+^, Fe^2+^	200	<5.0

The reproducibility of the proposed sensor (AuNPs/en-MWCNTs/SPE) for Amoxicillin detection was evaluated by determining Amoxicillin (10 µM) with six separate sensors. The result shows that all the sensors exhibit almost similar response with relative standard deviation (RSD) of 1.9% suggesting that the proposed sensor is reproducible during reproduction. Repeatability of the proposed sensor gave RSD of 3.9% for five successive measurements using same electrode suggesting that the fabricated sensor can be used for more measurements. Stability of the proposed sensor was investigated by preparing different electrodes and stored at room temperature and under sun. The current response towards determination of 10 µM Amoxicillin at regular intervals (per day) for a period of one week was evaluated and the sensor retained 90% of its initial signal suggesting good stability at room temperature or under the sun within a week.

### 3.4. Application of the Proposed Method in Real Sample Analysis

The applicability of the proposed sensor was evaluated by analyzing Amoxicillin in bovine milk samples (Putra Mart, UPM and Fresh Milk, Johor Malaysia). The samples were prepared according to the procedure described in Section 2.3. Each sample was spiked with different concentrations of Amoxicillin. As can be seen (Table 3), good recoveries were reported ranging from 91% to 95%. These suggest that the matrix of the samples has no significant effect on the voltammetric determination of Amoxicillin at the modified electrode. In addition, the results of fabricated sensor were compared with standard test method for amoxicillin (ASTM) [34]. The result shows that the fabricated sensor can serve as an alternative procedure for determination of Amoxicillin in milk samples with simple pretreatment, high sensitivity, good selectivity and economic advantages.

**Table 3:** Determination of amoxicillin in some bovine milk samples using fabricated sensor. ($\bar{X}\pm SD,\;n=3)$.

Sample	Spiked (µM)	Found (µM)	Recovery (%)	HPLC
Putra Mart Milk ^1^	-	<LOD	-	ND ^2^
	1.0	0.940 ± 0.011	94.	0.978 ± 0.058
	2.0	1.91 ± 0.023	95.5	1.976 ± 0.35
Fresh Milk ^3^	-	<LOD	-	ND
	1.0	0.915 ± 0.038	91.5	0.980 ± 0.15
	2.0	1.87 ± 0.060	93.5	1.957 ± 0.01

^1^ Universiti Putra Malaysia, Serdang, Malaysia; ^2^ Not detected; ^3^ Johor Farms, Johor, Malaysia.

## 4. Conclusions

In this study, a novel voltammetric sensor was constructed for the detection of Amoxicillin residue in bovine milk using AuNPs/en-MWCNTs nanocomposites. The fabricated electrode exhibits lower detection limit, wide linear dynamic range, good repeatability, reproducibility, and stability as well as being free from the effects of common interfering species in analysis of amoxicillin residue in bovine milk. The combination of good electron transfer property of MWCNTs and the catalytic property of AuNPs are responsible for enhancement of electrocatalytic performance of the SPE. The constructed sensor is sensitive, selective and economical with short response time. The result obtained in the analysis of Amoxicillin in spiked bovine milk samples demonstrated the applicability of this sensor for analysis of food samples. 

## Supplementary Materials

The following are available online at www.mdpi.com/link: Figure S1: FTIR Spectra of (A) Pristine MWCNTs (B) Acid treated MWCNTs and (C) Amine treated functionalized MWCNTs. Figure S2: The relationship between pH and oxidation peak potential of Amox (30 µM) on the surface of AuNPs/en-MWCNTs/SPE. Figure S3: Effect of drop casting volume of AuNPs/en-MWCNTs nanocomposite on the oxidation peak current of 30 µM Amox in the presence of 0.1 M Phosphate buffer (pH 7.0), accumulation time 180 s, accumulation potential −0.4 V, scan rate 0.1 V·s^−1^. Figure S4: Effect of HAuCl4 concentration on the oxidation peak current of 30 µM Amox in the presence of 0.1 M PBS (pH 7.0), accumulation time 180 s, accumulation potential −0.4 V, scan rate 0.1 V·s^−1^.
