# Supplementary material for: Construction of an Electrochemical Sensor Based on Carbon Nanotubes/Gold Nanoparticles for Trace Determination of Amoxicillin in Bovine Milk
Source: Sensors (Basel). 2016 Jan 20;16(1):56. doi: 10.3390/s16010056 (PMC4732089; doi:10.3390/s16010056)
Supplement: Supplementary file 1 [file sensors-16-00056-s001.pdf]

Supplementary Materials: Construction of an Electrochemical Sensor Based on Carbon Nanotubes/Gold Nanoparticles for Trace Determination of Amoxicillin in Bovine Milk

Aliyu Muhammad, Nor Azah Yusof, Reza Hajian and Jaafar Abdullah

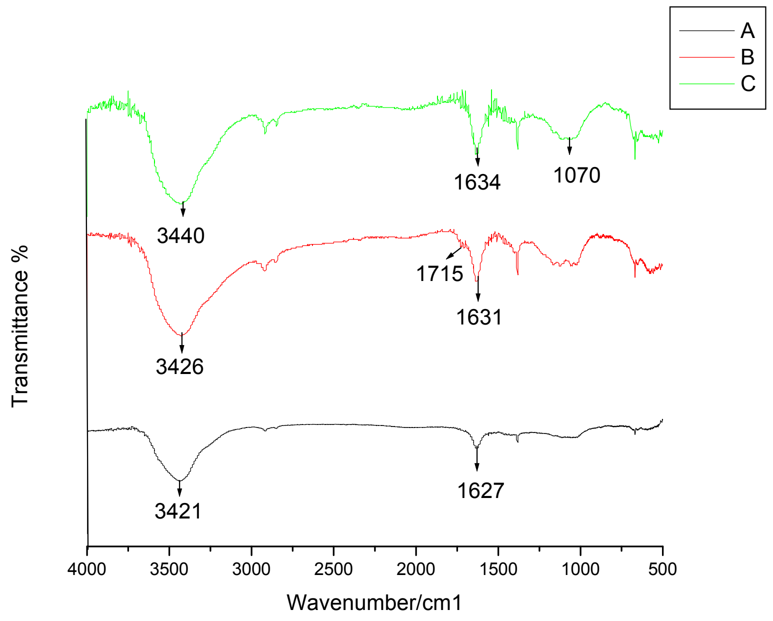

Figure S1. FTIR Spectra of (**A**) Pristine MWCNTs; (**B**) Acid treated MWCNTs; and (**C**) Amine treated functionalized MWCNTs.

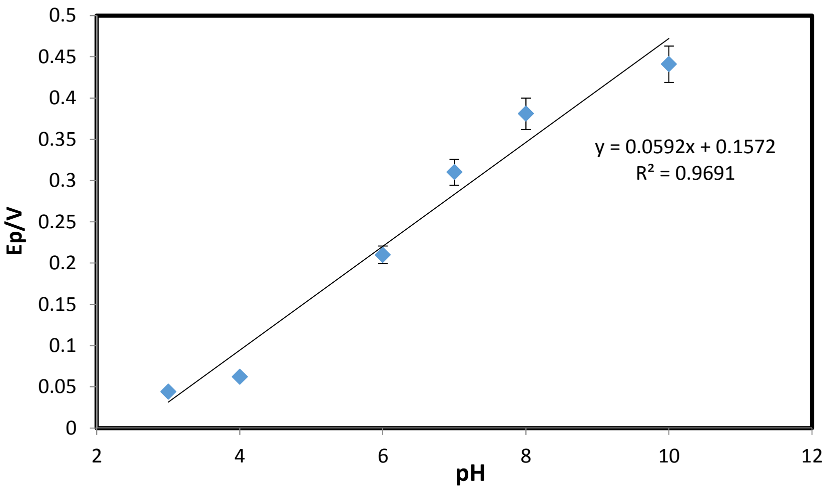

Figure S2. The relationship between pH and oxidation peak potential of Amox (30 μM) on the surface of AuNPs/en-MWCNTs/SPE.

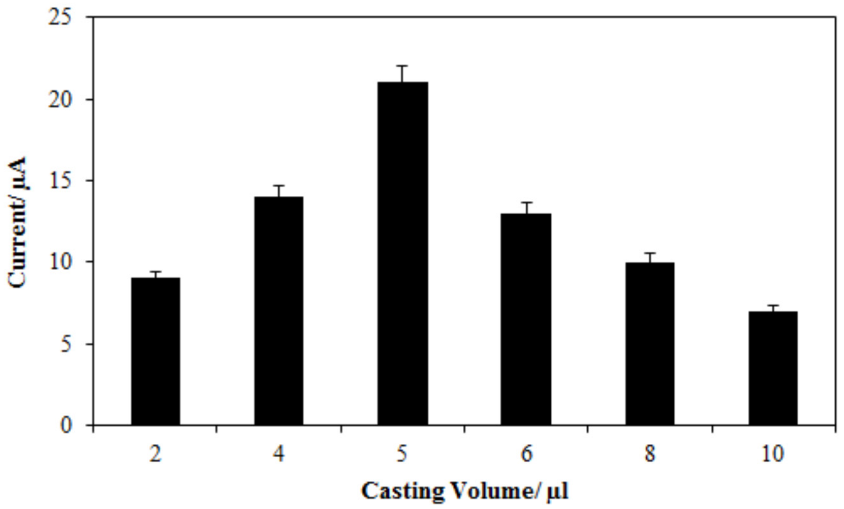

Figure S3. Effect of drop casting volume of AuNPs/en-MWCNTs nanocomposite on the oxidation peak current of 30 µM Amox in the presence of 0.1 M Phosphate buffer (pH 7.0), accumulation time 180 s, accumulation potential −0.4 V, scan rate 0.1 V·s^{-1}.

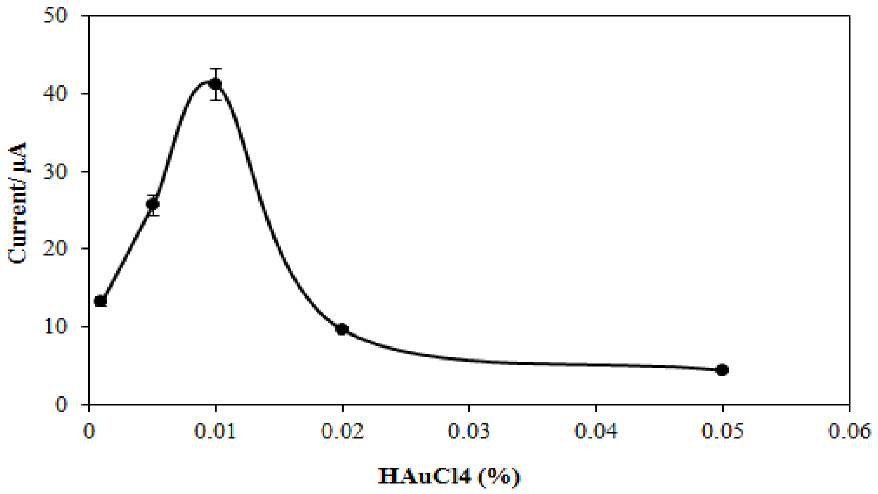

Figure S4. Effect of HAuCl4 concentration on the oxidation peak current of 30 µM Amox in the presence of 0.1 M PBS (pH 7.0), accumulation time 180 s, accumulation potential −0.4 V, scan rate 0.1 V·s^{-1}.
